# Endometrial Cancer: Molecular and Cellular Basis of Tumor Development, Novel Biomarkers and Therapeutic Agents, and Innovative Research Approaches

**DOI:** 10.1155/2014/710405

**Published:** 2014-01-16

**Authors:** Donghai Dai, Andrew P. Bradford, Eric R. Prossnitz

**Affiliations:** ^1^Department of Obstetrics and Gynecology, University of Iowa Carver College of Medicine, Iowa City, IA 52242, USA; ^2^Department of Obstetrics and Gynecology, University of Colorado Health Science Center, Denver, CO 80045, USA; ^3^Department of Cell Biology and Physiology, University of New Mexico, Albuquerque, NM 87131, USA

Carcinoma of the endometrium is the most common cancer of the female reproductive tract with over 40,000 new cases diagnosed per year and over 7,000 deaths per year in the United States alone. Although a majority of endometrial tumors present with well-differentiated low grade endometrioid histology (Type 1), expressing high levels of estrogen and progesterone receptors (ER/PR) as well as epidermal growth factor receptor (EGFR), about one-quarter present as more advanced and aggressive tumors (Type 2), that are unlikely to be ER/PR-positive and have a much poorer prognosis. Although histology, genetic aberrations, and epidemiological profiles overlap between the two tumor types, they appear to represent distinct carcinogenic processes with distinct molecular characteristics. Whereas type 1 tumors are typically preceded by endometrial hyperplasia and are associated with a loss of PTEN expression as well as abnormalities in *β*-catenin, Kras, and DNA mismatch repair genes, type 2 tumors represent a heterogeneous group of tumors including high-grade (undifferentiated) endometrioid carcinomas, uterine papillary serous carcinomas, clear cell carcinomas, and carcinosarcomas. Whereas, uterine papillary serous carcinomas are typically associated with p53 mutations and often Her-2/neu mutations, with PTEN mutations being rare, carcinosarcomas, characterized by both malignant epithelial and mesenchymal components, are associated with many of the epidemiological risk factors linked to endometrioid carcinomas including obesity and tamoxifen therapy, which suggests that dysregulated estrogen signaling may have a role in its pathogenesis and may represent a therapeutic target. In this special issue on endometrial cancer, papers address not only molecular and cellular aspects of endometrial cancer formation but also novel studies of biomarkers as well as potential new therapeutic agents and approaches, which together could have great impact on the diagnosis and treatment of women with endometrial cancer.

The first paper of this issue examines the expression of Placenta-specific protein 1 (PLAC1), a small, secreted protein normally expressed only in trophoblast cells in the mammalian placenta. E. I. Devor and K. K. Leslie demonstrate that PLAC1 is ubiquitously expressed in tumors originating from the uterine epithelium and that expression is higher in more advanced and aggressive endometrial serous adenocarcinomas and carcinosarcomas. In the second paper, A. M. Thorne and colleagues demonstrate that expression of active, myristoylated PKC*α* confers ligand-independent activation of estrogen receptor-dependent promoters and enhances responsiveness to estrogen, suggesting that PKC*α* signaling, possibly via PI3K/Akt, may be a critical component of the hyperestrogenic environment with activation of ER that may underlie the development of estrogen-dependent endometrial hyperplasia and malignancy.

In the third paper, H. E. Dinkelspiel and colleagues discuss both risk factors for and protective factors against the development of endometrial cancer as well as primary and alternative management options for this disease. In the fourth paper, K. K. Leslie and colleagues highlight new information linking the expression of the estrogen receptors (both *α* and *β*) to outcome, discussing the value of employing ER as a biomarker for positive outcome and hormonal treatment. The fifth paper in this issue examines the feasibility of RNA and DNA extraction from fresh Pipelle and archival endometrial tissues for use in gene expression and SNP assays, revealing that fresh frozen Pipelle samples, which are minimally invasive, yield excellent quantity and quality of RNA for gene expression arrays.

In the sixth paper of this special issue, S. Nair and colleagues demonstrate that adipocytes have potent proliferative paracrine effects on endometrial cells, which are in part mediated by TNF, since the proliferative effects of adipocyte-conditioned medium could be reversed by anti-TNF antibodies. In the seventh paper, W. K. Petrie and colleagues examine the mechanisms of estrogen signaling in ER-negative endometrial cancer cells. They demonstrate that estrogen, as well as SERMs and SERDs, such as tamoxifen, fulvestrant and raloxifene, continues to activate multiple signaling pathways in the absence of ER through the G protein-coupled estrogen receptor GPR30/GPER and that estrogen-stimulated ER-negative endometrial tumor growth is blocked by a selective GPER antagonist. Finally, in the last paper, X. Meng and colleagues test whether synthetic lethality can be achieved in endometrial cancer cells expressing mutant p53 by combining paclitaxel with agents to overcome G2/M arrest thereby inducing mitotic catastrophe. They reveal that synthetic lethality could be generated by combining paclitaxel with BIBF1120 (an investigational VEGFR, PDGFR, and FGFR multityrosine kinase inhibitor with established antiangiogenic activity), which together abrogated the G2/M checkpoint in p53-null endometrial cancer cells via modulation of G2/M checkpoint regulators followed by induction of mitotic cell death. Conversely, in endometrial cancer cells expressing an oncogenic gain-of-function p53 mutation, synthetic lethality was induced by combining paclitaxel with BIBF1120 and a histone deacetylase inhibitor, which served to destabilize mutant p53.

Basic and preclinical studies based on an improved understanding of the molecular and cellular aspects of tumor development and progression as described in this special issue will continue to serve as important approaches for the development of new biomarkers for diagnosis and prognosis as well as the innovative design of novel clinical trials utilizing molecularly targeted therapeutics.

*Donghai Dai* *Donghai Dai* *Andrew P. Bradford* *Andrew P. Bradford* *Eric R. Prossnitz* *Eric R. Prossnitz*

